# The Cost-Effectiveness of Reclassification Sampling for Prevalence Estimation

**DOI:** 10.1371/journal.pone.0032058

**Published:** 2012-02-13

**Authors:** Airat Bekmetjev, Dirk VanBruggen, Brian McLellan, Benjamin DeWinkle, Eric Lunderberg, Nathan Tintle

**Affiliations:** 1 Department of Mathematics, Hope College, Holland, Michigan, United States of America; 2 Department of Mathematics, Statistics and Computer Science, Sioux Center, Dordt College, Iowa, United States of America; Genentech, United States of America

## Abstract

**Background:**

Typically, a two-phase (double) sampling strategy is employed when classifications are subject to error and there is a gold standard (perfect) classifier available. Two-phase sampling involves classifying the entire sample with an imperfect classifier, and a subset of the sample with the gold-standard.

**Methodology/Principal Findings:**

In this paper we consider an alternative strategy termed reclassification sampling, which involves classifying individuals using the imperfect classifier more than one time. Estimates of sensitivity, specificity and prevalence are provided for reclassification sampling, when either one or two binary classifications of each individual using the imperfect classifier are available. Robustness of estimates and design decisions to model assumptions are considered. Software is provided to compute estimates and provide advice on the optimal sampling strategy.

**Conclusions/Significance:**

Reclassification sampling is shown to be cost-effective (lower standard error of estimates for the same cost) for estimating prevalence as compared to two-phase sampling in many practical situations.

## Introduction

Disease prevalence estimates in the presence of an imperfect classifier (e.g. a diagnostic test with sensitivity and/or specificity less than one) are biased. The extent of this bias depends on the true prevalence of the disease and the sensitivity and specificity of the classifier under consideration. When a “gold standard” (that is a classifier that has both sensitivity and specificity equal to one) is available, at least two sampling strategies have been proposed in order to achieve unbiased prevalence estimates. The first option (one-phase sampling) involves classifying all individuals in the sample using the gold standard. However, when the gold standard is significantly more expensive than an imperfect classifier, two-phase sampling may be cost-effective [1]–[6]. Two-phase (or “double”) sampling involves classifying all individuals in the sample with the imperfect mechanism, and then reclassifying a subset of individuals in the sample with the gold standard. Essentially, two-phase sampling allows the investigator to estimate the sensitivity and specificity of the imperfect classifier using individuals in the sample who have been classified by both the gold standard and the imperfect classifier. These estimates can then be used to adjust the prevalence estimate to be unbiased.

An alternative strategy to two-phase sampling is reclassification sampling. In this design, the entire sample is classified with the imperfect classifier, followed by a random subset of the sample classified a second time with the imperfect classifier; generalizations of reclassification sampling allow for individuals to be classified any number of times by the imperfect classifier. Reclassification sampling was first proposed by Sutcliffe in 1965 and soon after by Koch (1969) [7]–[9]. Since then, several articles have considered reclassification sampling (see Fujisawa and Izumi [10] for a brief review), however, these articles consider situations where some portion of the sample is classified at least three times.

More recently, a hypothesis test for association between two categorical variables was proposed for reclassification sampling (applied to Single Nucleotide Polymorphism (SNP) genotype and disease phenotype data) [11]–[13]. In that setting one of the variables is measured perfectly, the other variable is measured imperfectly, and some fraction *r* of individuals is reclassified on the imperfectly measured variable. It was shown that, as long as classification errors are independent between classifications, only two classifications are needed in order to carry out the hypothesis test of association.

In this paper we explore practical situations that can provide estimates of prevalence if individuals are only classified twice. Further, we provide a cost-effectiveness analysis of reclassification sampling and compare it with one- and two-phase sampling. Specifically, we evaluate which sampling strategy is the most cost-effective in terms of the variance of the prevalence estimate and show that reclassification sampling is cost-effective in many practical situations. Throughout this paper we use the term “disease prevalence” however, the results generalize to any binary classification procedure that makes independent errors.

## Materials and Methods

### Sampling strategy

We consider a sampling strategy where a fraction of the original sample (denoted *r*) is classified exactly twice using an imperfect classifier. The remaining fraction of the sample, *1-r*, is, therefore, classified exactly once using the same classifier. One of the goals of this paper is to find an optimal value for *r*. We note that all individuals are classified into one of two mutually exclusive groups, which for convenience we call “Diseased” (Group 1) and “Not Diseased” (Group 2).

### Error Assumptions

We make a common assumption (e.g. Fujisawa and Izumi [10]) that classification errors have a constant probability for all sample units. Also, we assume that classification errors are independent, meaning individuals who were misclassified the first time are as likely as anyone else to be misclassified the second time they are classified. For example, consider an individual who happens to be in the 3% of individuals misclassified the first time they were classified. If this individual is classified a second time, the independent error assumption says that this individual still has a 3% chance of being misclassified.

### Notation

*y* = the total number of individuals in the sample that are classified exactly once.

*z* = the total number of individuals in the sample that are classified exactly twice.

*N = y+z = *the total number of individuals in the sample.

*r = z/N = *the fraction of the sample that is classified twice, where 0≤*r*≤1.

*y_i_* = among individuals who are classified exactly once, the number of individuals who are classified to the *i^th^* group (*i* = 1,2).

*z_ij_* = the number of individuals classified exactly twice who are classified to the *i^th^* (*i* = 1,2) group once, and the *j^th^* (*j* = 1,2) group once. Therefore, if 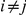 then the individual has been inconsistently classified.

*ε_ij_* = the probability that an individual who actually belongs in the *i^th^* category is classified to the *j^th^* category, where for *i* = 1 or *i* = 2, 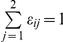. If *i≠j* then *ε_ij_* is the probability of a classification error. We let *i* = 1 be “diseased“ and *i* = 2 be “not diseased“ and so *ε_11_* represents sensitivity and *ε_22_* represents specificity of the test.

*p_i_* = the true probability that an individual actually belongs in the *i^th^* category, where 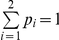. Thus, *p_1_* represents the true population prevalence of the disease.

p^*^_i_ = the proportion of observed individuals in the *i^th^* category after a single classification.

p^*^_ij_ = the proportion of observed individuals in the *i^th^* group once and the *j^th^* group once. We note that if there are no classification errors (i.e. sensitivity = specificity = 1), then p*_ii_ = p*_i_ = p_i_ and, for all *i≠j*, p^*^_ij_ = 0.

We also briefly introduce the parameters related to budget and cost, which are considered in section titled *Optimal sampling strategy for prevalence estimation* in the *Results*.

*c* = the cost per person of the imperfect classifier,

*a* = the cost per person for acquisition or enrollment in the study.

*c_g_* = the cost per person of the gold standard classifier.

*B* = the total budget available for sample acquisition and classification.

## Results

### Estimating prevalence (*p_1_*) using two classifications

System (*1*) describes the relationship between parameters if there are only two classifications.
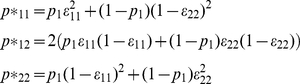
(1)These equations are not independent due to the constraint 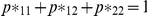 and, hence, the system is not uniquely solvable. To resolve that problem, one can either reduce the number of parameters or add an equation to the system. Fujisawa and Izumi [10], as well as Sutcliffe [6], [7], introduce additional equations by requiring at least three classifications in order to estimate prevalence, sensitivity and specificity. It is possible, however, to reduce the number of parameters in the system with an alternative constraint and avoid a third classification. We assume that there is a relationship between sensitivity and specificity. For example, we might assume that *ε_22_* is 80% of *ε_11_*, or, in the simplest case, that *ε_22_ = ε_11_*. In this paper we consider the following functional relationship, *ε_22_ = θ ε_11_*, where *θ* can be any positive number as long as 0≤*ε_22_*≤*1*. In this paper we consider the robustness of estimation and, ultimately, optimal sampling strategy decisions if the value of *θ* is incorrect.

If we assume that *θ* is known, we can rewrite *p_1_, ε_11_* and *ε_22_* as functions of p*_11_ and p*_12_. (See *Text S1* for details).
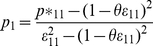
(2)

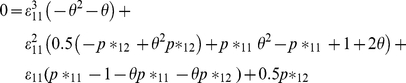
(3)Equation (3) can be solved using the cubic formula. Since p*_ij_ (*i,j = 1,2*) in (*1*) have a multinomial distribution, we know that their MLE's are the observed counts in each cell of the multinomial distribution divided by the sample size (e.g., 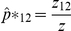, where 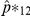 is the MLE of p*_12_). The system of equations (2), (3) can be significantly simplified if we consider *θ* = 1. In this case, by the invariance property of MLE's [14], we get
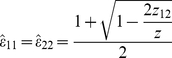
(4)

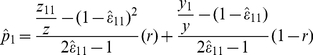
(5)Where 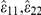 and 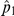 are the MLE's of *ε_11_, ε_22_*, and *p_1_*, respectively. Note that equation (*5*) combines information from individuals reclassified once and individuals reclassified two times (See *Text S1* for details). Later, in the section titled *Robustness of the model assumptions (case θ≠1)*, we consider the robustness of this approach for other values of *θ*.

In order to find the expected value and variance of the prevalence estimate (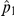) given in (*5*) we use a first order Taylor series approach as described in Casella and Berger [14]. The Taylor series approach says for a set of random variables 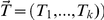 with means 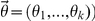 that for any differentiable function 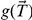, 
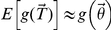
. In our case, the functions used for 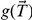 are equations shown in *Text S1*, and thus we have the result that 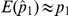, 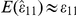 and 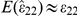. We can use the same Taylor series approach to find the variance of 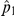 *as*

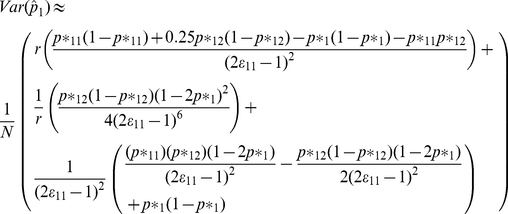
(6)
*Text S2* provides details on how the variance formula is derived and also provides the variance of the sensitivity estimate 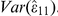


A simulation study using R [15] was conducted in order to investigate the quality of the Taylor series approximation. To conduct the simulation, seven values of disease prevalence (0.001, 0.01, 0.05, 0.10, 0.25, 0.40 and 0.50), five values of sensitivity (equal to specificity; 0.80, 0.90, 0.95, 0.99, 0.999), ten values of the reclassification rate *r* (0.01, 0.05, 0.10, 0.25, 0.50, 0.75, 0.90, 0.95, 0.99, 1.0), and three values for the total sample size *n* ( = *z+y*; 500, 1000 and 5000) were selected. We considered all possible combinations of these parameters (7×5×10×3 = 1050), except for 105 settings where z = r*n<50, since estimates with less than 50 reclassified individuals can be unstable. For each of the 945 (1050-105) combinations examined, 2000 samples were simulated.

Ninety-two percent (868/945) of the cases examined had a simulated expected value within 10^−3^ of the true sensitivity with 100% of the cases (945/945) within 10^−2^. Similarly, 79% (749/945) of the cases examined had a simulated expected value within 10^−3^ of the true prevalence with 98% (922/945) of the cases within 10^−2^. The most biased estimates occurred when prevalence was very low (e.g., prevalence≤0.01) and when sensitivity/specificity was lower (e.g., sensitivity≤0.90). In these cases, the tendency was to underestimate the sensitivity/specificity, which results in an overestimate of the prevalence. For example, the most biased sensitivity estimate was when the true sensitivity was 80%, but the estimate was 79.4%, which occurred when n = 5000 and r = 0.01, for prevalence 10%. Additionally, the most biased prevalence estimate was when prevalence was 0.1%, sensitivity was 80%, r = 0.05 and n = 1000, when the estimated prevalence was 3.0%.

To ease in interpretation, and to allow for comparison across different sample sizes, variance differences between the simulated and theoretical variance are reported multiplied by a factor of *1/n*.

Eighty-five percent (799/945) of the cases examined have a difference between theoretical and simulated variance of less than 10^−1^ *(1/n)* for sensitivity with 96% (911/945) less than *1/n*. Also, 65% (615/945) of the cases have a difference between theoretical and simulated variance of less than 10^−1^ *(1/n)* for prevalence with 87% (826/945) having a difference in variances less than *1/n*. Similar to the results for expected value, the most biased estimates occurred when prevalence was very low (e.g., prevalence≤0.01) and when sensitivity/specificity was lower (e.g., sensitivity≤0.90). In these cases, the simulated variance tended to be more than theoretical (predicted) variance for sensitivity, and less than the theoretical (predicted) variance for prevalence. For example, the most biased variance estimate for sensitivity occurred for sensitivity 80%, prevalence 10%, r = 5%, n = 1000, when the simulated variance was 0.0004 larger than the theoretical (predicted) variance. Additionally, the most biased estimate of the variance for prevalence occurred when the prevalence was 0.1%, sensitivity was 80%, r = 0.01 and n = 5000, when the simulated variance was 0.007 less than the theoretical (predicted) variance.

As anticipated, the Taylor Series approximation approach provides reasonable estimates, except in situations where the most extreme values of the parameters occur. Having established that the estimates shown in (*4*) and (*5*) are approximately unbiased with known variance, confidence intervals are easily obtained using the delta method [14]. Details of a simulation study which verified proper coverage probabilities for the confidence intervals are not shown.

### Optimal sampling strategy for prevalence estimation

We now discuss how to optimize reclassification sampling, and then compare an optimally designed reclassification sampling study to the traditional one- and two-phase sampling methods.

In order to optimally design a reclassification sampling study we need to establish the value of the reclassification rate *r*, *0<r≤1*, so that the variance of the 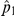 estimator (given in (*6*)) is minimized for a fixed budget *B*. The available budget is used to cover costs of sample acquisition (*Na*), as well as initial and subsequent classification (*2cNr+N(1−r)c*), leading to equation (*7*):
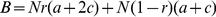
(7)Based on equations *(6)* and *(7)* we find the variance of the prevalence estimate as a function of *r*. We can then find the optimal value of *r* by finding the minimum variance of the prevalence estimate 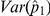 for *0<r≤1*.

We examined six different values of *p_1_* (0.01, 0.05, 0.10, 0.25, 0.40 and 0.50), seven different values of *ε_11_* (0.999, 0.99, 0.98, 0.95, 0.90, 0.85, 0.80), nine different values of *c* (0.001, 0.01, 0.05, 0.10, 0.20, 0.50, 1, 2, and 5), and two different values of *a* (0 and 1; representing samples that have already been obtained (*a* = 0), or samples that have acquisition cost that can be expressed relative to the classification cost, *c* (*a* = 1) yielding 756 total combinations. While the budget (and hence sample size) does affect the value of the variance, it does not change the optimal value of *r* (see *Text S3* for details).

Overall, 379 of the 756 optimal values of *r* were at 1.0, and 109 times the optimal value of *r* was at 0.01. We used *r* = 0.01 as the minimum value of *r*. The remaining 256 cases yielded an optimal *r* between 0.01 and 1. In 100 of the 109 times that the optimal value was at 0.01, the prevalence was 50% (the other nine times was when prevalence was at 40%). In contrast, when the optimal reclassification rate was at *r = 1*, prevalence tended to be lower, acquisition costs were present, classification costs were low, and sensitivity was lower.

As is described in the introduction, previous work by McNamee [3] compared the cost effectiveness of two-phase (double) sampling to one-phase sampling. Text S*4* uses our notation to give equations for the variance of one and two-phase sampling.

To compare reclassification sampling to one- and two-phase sampling we first establish which of one- or two-phase sampling is the most cost-effective by minimizing the two variances given in *Text S4* equations (*S.4.1*) and (*S.4.2*). Then we compare the variance obtained from an optimally designed reclassification study to the minimum of the other two. *Tables* 1, 2, 3 how the ratio of 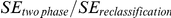 for the prevalence estimate. In all cases where the ratio is greater then 1, reclassification sampling provides a smaller standard error for the same budget. The cost ratio is the ratio of the cost of the gold standard (*c_g_*) to the classification cost for the cheap classifier (*c*). All values shown in *Tables 1* *,* *2* *,* *3* assume an acquisition cost (*a*) of 0, though values for *a* = 1 follow a similar pattern (detailed results not shown). Additionally, as explained in *Text S4*, values in *Tables 1* *,* *2* *,* *3* are independent of budget/sample size considerations.

**Table 1:** Ratios of Standard Errors (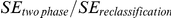) when Sensitivity = Specificity = 0.99.

		Cost Ratio (*c_g_/c*)				
Prevalence	Optimal *r* a	5	25	50	100	500
0.01	1	0.88	1.19	1.47	1.87	3.59
0.05	0.32	1.04	1.46	1.78	2.22	4.11
0.10	0.20	1.15	1.61	1.95	2.43	4.47
0.25	0.08	1.29	1.78	2.16	2.69	4.92
0.40	0.03	1.36	1.88	2.27	2.83	5.17
0.50	0.01	1.39	1.93	2.33	2.90	5.30

a *The optimal reclassification rate for reclassification sampling.*

**Table 2:** Ratios of Standard Errors (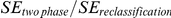) when Sensitivity = Specificity = 0.95.

		Cost Ratio (*c_g_/c*)				
Prevalence	Optimal *r* a	5	25	50	100	500
0.01	1	0.94	1.54	2.04	2.78	5.94
0.05	1	1.09	1.86	2.45	3.28	6.79
0.10	0.74	1.16	1.96	2.56	3.40	6.98
0.25	0.21	1.41	2.34	3.03	4.01	8.13
0.40	0.07	1.58	2.60	3.37	4.45	9.01
0.50	0.01	1.68	2.76	3.57	4.71	9.54

a *The optimal reclassification rate for reclassification sampling.*

**Table 3:** Ratios of Standard Errors (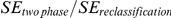) when Sensitivity = Specificity = 0.80.

		Cost Ratio (*c_g_/c*)				
Prevalence	Optimal *r* a	5	25	50	100	500
0.01	1	0.33	0.65	0.90	1.26	2.78
0.05	1	0.68	1.33	1.84	2.56	5.61
0.10	1	0.87^b^	1.72	2.36	3.28	7.13
0.25	1	1.08^b^	2.19	2.99	4.12	8.88
0.40	0.22	1.19^b^	2.44	3.33	4.57	9.84
0.50	0.01	1.34^b^	2.75	3.74	5.14	11.06

a *The optimal reclassification rate for reclassification sampling.*

b *Compared to one-phase sampling, because in these cases, one-phase sampling provides estimates with smaller standard errors then with two-phase sampling.*

*Tables 1* *,* *2* *,* *3* present values for a variety of prevalence, sensitivity and cost ratio values. We note that in many cases, reclassification sampling provides a substantial reduction in the standard error of the prevalence estimate as compared to one or two-phase sampling. As shown in *Tables 1* *,* *2* *,* *3*, reclassification becomes increasingly effective as the cost ratio increases (that is, the gold standard becomes more expensive as compared to the imperfect classifier). Also, reclassification sampling provides increasing advantages as the prevalence increases.

### Robustness of the model assumptions (case *θ≠1)*

It is of interest to know how robust the estimates provided earlier ((*4*) and (*5*)) are to violations of the assumption that *θ = 1*. To answer this question we conducted a simulation study to evaluate the bias in cases where sensitivity and specificity are not equal. We extended an earlier simulation study and examined seven values of disease prevalence (0.001, 0.01, 0.05, 0.10, 0.25, 0.40 and 0.50), five values of sensitivity (0.80, 0.90, 0.95, 0.99, 0.999), five values of specificity (0.80, 0.90, 0.95, 0.99, 0.999), ten values of the reclassification rate *r* (0, 0.01, 0.05, 0.10, 0.25, 0.50, 0.75, 0.90, 0.95, 0.99, 1.0), and three values for the total sample size *n* ( = *z+y*; 500, 1000 and 5000). For each combination of prevalence, sensitivity, specificity, reclassification rate and sample size (4725, since we eliminated 525 combinations where z = r*n<50), 2000 samples were simulated.

In order to investigate robustness, we started by evaluating the extent of bias for prevalence estimates in cases where 0.95≤*θ*≤1.05, but *θ*≠1. In 73.5% (695/945) of cases, the bias for the prevalence estimate was within 1% of the true prevalence, with all bias within 3% of the true prevalence estimate. However, in contrast to our results earlier for cases where *θ* = 1, the largest bias occurred when the prevalence was large. For example, the largest bias occurred when prevalence was 50%, sensitivity was 99.9%, specificity was 95%, n = 500, r = 0.25, and the average observed prevalence was 53.7%.

As *θ* deviated more and more from one, the bias increased rather dramatically, The maximum bias observed was 12.6% (estimated prevalence of 52.6%), when the observed prevalence was 40%, the sensitivity was 0.999, the specificity was 80%, r = 5%, and n = 1000. Thus, estimates of prevalence are relatively robust in situations where the sensitivity and specificity are not equal (*θ*≠1), as long as the extent of the inequality keeps 0.95≤*θ*≤1.05.

In the previous section we evaluated the bias of the prevalence estimates to misspecifications of *θ*. In this section we consider the robustness of the ratio of standard errors comparing two-phase to reclassification sampling (presented in *Tables 1* *,* *2* *,* *3*) to misspecifications of *θ*. To do this we compare the simulated standard error of the prevalence estimate to the theoretical standard error of the prevalence estimate (for a value of *θ* equal to 1). We recommend a conservative approach where a researcher should use the value of specificity for both parameters *ε_11_* and *ε_22_* in the theoretical computation as long as the prevalence is less than 50% and when prevalence is more than 50% use the sensitivity as a value for *ε_11_* and *ε_22_*. For example, if sensitivity = 90%, specificity = 95% and prevalence is less than 50%, the researcher should use *ε_11_* = *ε_22_* = 0.95 in the theoretical computation. Using this rule in the theoretical computation yields theoretical ratios of 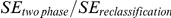 within 10% of the ratios presented in *Tables 1* *,* *2* *,* *3* (meaning (Observed Ratio-Theoretical Ratio)/Theoretical Ratio is no more than 0.1) in 98.6% of cases examined as long as the expected values of *z_11_* and *z_12_* are both at least 5 and 0.90≤*θ*≤1.10. Thus, we have shown that the ratios of standard errors in *Tables 1* *,* *2* *,* *3*are relatively robust to situations where the sensitivity and specificity are not equal.

### Application of reclassification sampling

Fujisawa and Izumi [10] provide prevalence, sensitivity, and specificity estimates based on repeated classifications of an individual's blood type according to the MNSs blood typing system. As a proof of concept of the methods proposed earlier for computing estimates and confidence intervals for sensitivity, specificity and prevalence we apply the estimation procedure to data from Fujisawa and Izumi [10] on individuals only classified two times. Results are shown in *Table 4*. These estimates were computed using software available at (http://www.dordt.edu/statgen and following the links to software).

**Table 4:** Prevalence estimation using reclassified data from Fujisawa and Izumi (2000).

	Number of subjects testing positive for the antigen *M*					
City	*z_11_*	*z_12_*	*z_22_*	*n = z*	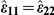 (95% CI)	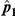 (95% CI)
Hiroshima	1918	8	419	2345	0.998 (0.997, 0.999)	0.821 (0.805, 0.836)
Nagasaki	958	13	257	1228	0.994 (0.992, 0.998)	0.788 (0.766, 0.811)

Our estimates of prevalence and sensitivity/specificity are within the confidence intervals provided by Fujisawa and Izumi, except for the specificity confidence interval provided by Fujisawa and Izumi for Hiroshima (0.957, 0.993), which does not include our point estimate of 0.998.

## Discussion

In this paper we evaluated reclassification sampling, considering the situation where some fraction of the sample is classified by the same imperfect method two times. We demonstrated how to estimate prevalence and sensitivity/specificity for reclassification sampling. We established that reclassification sampling is cost-effective in many cases when compared to one and two-phase sampling. We also demonstrated the extent of robustness of estimates and the sampling strategy decision to violations of model assumptions.

The fact that reclassification sampling is more cost-effective than one- and two-phase sampling to estimate prevalence may not be intuitive. However, consider the following example. Let's assume that a diagnostic test with sensitivity and specificity of 95% is available for $1.00 per application, and a gold-standard diagnostic test is available for $100. *Table 2* shows that if the prevalence of the disease in the population is 1% then reclassification sampling is approximately 2.78 times more cost-effective than two-phase sampling. Using optimality criterion for two-phase sampling, a researcher will use the gold standard on approximately 11.5% of the total sample, *n*. For a budget of $10,000 this means that a researcher will be able to have approximately 800 people in the study. An optimally designed reclassification study uses an *r = 1* (everyone gets reclassified). Thus, the reclassification study will have 5,000 people in the study. In essence, having more than 6 times as many people in the reclassification study outweighs the perfect data obtained from the gold-standard in the two-phase sample.

The assumption of independent errors for the reclassification sampling strategy is crucial to its utility. If errors are not independent then reclassifying individuals does not “clean-up” the mistakes—instead misclassifying individuals time after time. There are likely many applications where the independent error assumption is legitimate. Tintle et al. [16] provide data which suggests that Single Nucleotide Polymorphism (SNP) genotyping errors appear to follow an independent misclassification pattern. Additionally, Fujisawa and Izumi [10] argue that the independent error assumption may be legitimate for blood typing data. Conceivably there are many other classification processes (diagnostic tests, etc.) where errors are independent and for which reclassification sampling provides an alternative, and in many cases more efficient, sampling strategy.

It is interesting to consider cases when the optimal strategy requires the reclassification of the entire sample (*r* = 1). It may suggest that increasing the number of reclassifications may provide further reduction in the variance estimate. We considered cases of multiple reclassifications when a separate optimal rate can be found for each stage. More specifically let 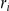 be the percentage of the sample that is reclassified *i* times. We considered the optimal selection of a vector of rates (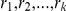) that minimizes 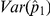. Note that having multiple classifications does not rely on knowledge of θ. However, a preliminary analysis of this design, using the EM-algorithm, did not reveal any substantial gains in the cost-effectiveness. In other words, three or more classifications provided little increase in efficiency as compared to two classifications.

We note that, in some settings, known values of the sensitivity and specificity are available. In these cases neither two-phase sampling nor reclassification sampling is necessary because prevalence estimates can be made unbiased by incorporating known sensitivity/specificity estimates into the estimation. The purpose of both two-phase sampling and reclassification sampling is to provide empirical estimates of sensitivity/specificity which can then be used to adjust prevalence estimates to be unbiased.

To this point, two-phase (double) sampling has been the primary alternative sampling strategy for investigators handling data subject to misclassification errors. McNamee [3] has shown that for prevalence estimation two-phase sampling can be cost-effective. However, reclassification can provide relatively large improvements in precision when compared to two-phase sampling with realistic and robust assumptions on sensitivity and specificity. Precision gains increase as the relative cost of the gold standard increases and as the prevalence increases. Software is provided to assist investigators in making a decision about which sampling strategy is most cost-effective based on their sampling costs, anticipated sensitivity/specificity and prevalence.

When two-phase sampling was originally proposed, the gold-standard classifier was used on a random subsample of all individuals. However, Cochran [17] and more recently McNamee [3] demonstrated how using the gold standard at different rates in different groups provides an even more optimal version of two-phase sampling. Conceivably, a similar concept could be applied to reclassification sampling. Specifically, rather than reclassifying a random subsample of all individuals, reclassify *r_1_* individuals who are diagnosed as “diseased” the first time, and reclassify *r_2_* individuals who are diagnosed as “not diseased” the first time, where *r_1_* is not necessarily equal to *r_2_*. Thus “conditional reclassification sampling” may provide an even further optimized reclassification sampling strategy. Preliminary simulation studies suggest this to be the case.

## Supporting Information

Text S1 **A general solution for finding estimates of sensitivity, specificity and prevalence, for any value of** ***θ*** **.**
(DOCX)

Text S2
**Finding the variance of** 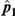 **and** 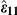 **if we assume that θ = 1.**
(DOC)

Text S3
**Finding the value of r on the range 0 to 1 that minimizes the variance of** 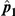.(DOC)

Text S4 **Variance for two-phase and one-phase sampling studies.**
(DOC)
