# Supplementary material for: The Cost-Effectiveness of Reclassification Sampling for Prevalence Estimation
Source: PLoS One. 2012 Feb 13;7(2):e32058. doi: 10.1371/journal.pone.0032058 (PMC3278465; doi:10.1371/journal.pone.0032058)
Supplement: Text S1 — A general solution for finding estimates of sensitivity, specificity and prevalence, for any value of θ . (DOCX) [file pone.0032058.s001.docx]

*Text S1*

This section shows a general solution for finding estimates of sensitivity, specificity and prevalence, for any value of *θ*.

The relation between known and unknown quantities is shown in (*S1.1*):

$\begin{matrix} p*_{11}=p_{1}\varepsilon_{11}^{2}+(1-p_{1})(1-\varepsilon_{22})^{2} \\ p*_{12}=2\left(p_{1}\varepsilon_{11}(1-\varepsilon_{11})+(1-p_{1})\varepsilon_{22}(1-\varepsilon_{22})\right) \\ p*_{22}=p_{1}(1-\varepsilon_{11})^{2}+(1-p_{1})\varepsilon_{22}^{2} \end{matrix}$ (*S1.1*)

We can solve the first equation in *(S1.1)* for$p_{1}$:$p_{1}=\frac{p*_{11}-(1-\varepsilon_{22})^{2}}{\varepsilon_{11}^{2}-(1-\varepsilon_{22})^{2}}=$ $\frac{p*_{11}-(1-\theta\varepsilon_{11})^{2}}{\varepsilon_{11}^{2}-(1-\theta\varepsilon_{11})^{2}}$ (*S1.2*)

where we assume that ε_22_= *θ* ε_11_.

Note that $\varepsilon_{11}^{2}-(1-\theta\varepsilon_{11})^{2}\neq0$ for all considered values of $\varepsilon_{11}$and $\theta$. By substituting the expression for $p_{1}$shown in (*S.1.2*) into the second equation of *(S 1.1)*, we obtain

$$p*_{12}=2\left(\frac{p*_{11}-(1-\theta\varepsilon_{11})^{2}}{\varepsilon_{11}^{2}-(1-\theta\varepsilon_{11})^{2}}\varepsilon_{11}(1-\varepsilon_{11})+\left(1-\frac{p*_{11}-(1-\theta\varepsilon_{11})^{2}}{\varepsilon_{11}^{2}-(1-\theta\varepsilon_{11})^{2}}\right)\theta\varepsilon_{11}(1-\theta\varepsilon_{11})\right)$$

Rewriting the previous equation in terms of $\varepsilon_{11}$, we obtain a cubic equation

$\begin{matrix} 0=\varepsilon_{11}^{3}\left(-\theta-\theta^{2}\right)+\varepsilon_{11}^{2}\left(0.5\left(-p*_{12}+\theta^{2}p*_{12}\right)+p*_{11}\theta^{2}-p*_{11}+1+2\theta\right)+ \\ \varepsilon_{11}(p*_{11}-1-\theta p*_{11}-\theta p*_{12})+0.5p*_{12} \end{matrix}$ *(S.1.3*)

Under condition $\theta=1$ equation *(S 1.3)* becomes

$0=-2\varepsilon_{11}^{3}+3\varepsilon_{11}^{2}+\varepsilon_{11}(-1-p*_{12})+0.5p*_{12}$ *(S 1.3’)*

We can factor out $(1-2\varepsilon_{11})$ since $\varepsilon_{11}=0.5$ is a root of *(S 1.3’)* and obtain the following equation

$$(1-2\varepsilon_{11})(\varepsilon^{2}_{11}-\varepsilon_{11}+0.5p^{*}_{12})=0$$

Since it is practical to assume that $\varepsilon_{11}$ *> 0.5*, we can directly solve it for $\varepsilon_{11}$:

$\varepsilon_{11}=\varepsilon_{22}=\frac{1+\sqrt{1-2p^{*}_{12}}}{2}$ *(S.1.4)*

In order to find an estimator for$p_{1}$ we consider a weighted combination of *p_1_* obtained from individuals classified exactly two times (denoted$p''_{1}$) and individuals classified exactly one time (denoted$p'_{1}$):

$$p_{1}=p''_{1}r+p'_{1}(1-r)$$

Here $p''_{1}=\frac{p*_{11}-(1-\varepsilon_{11})^{2}}{1-2\varepsilon_{11}}$ is the solution of *(S1.2)* and $p'_{1}=\frac{p*_{1}-(1-\varepsilon_{11})}{1-2\varepsilon_{11}}$ is the solution of equation

$p*_{1}=p_{1}\varepsilon_{11}+(1-p_{1})(1-\varepsilon_{22})$ (*S1.5*)
