# Supplementary material for: The Cost-Effectiveness of Reclassification Sampling for Prevalence Estimation
Source: PLoS One. 2012 Feb 13;7(2):e32058. doi: 10.1371/journal.pone.0032058 (PMC3278465; doi:10.1371/journal.pone.0032058)
Supplement: Text S2 — Finding the variance of and if we assume that θ = 1. (DOC) [file pone.0032058.s002.doc]

*Text S2*

In this section we find the variance of $\hat{p}_{1}$ and $\hat{\varepsilon}_{11}$ if we assume that *θ=1*. In order to find the variance of $\hat{p}_{1}$ *(6)* we use the Taylor Series method of approximation as described in Casella and Berger [14]. Details are shown below:

(*S2.1*)

Where

$p_{1}=\frac{p*_{11}-(1-\varepsilon_{11})^{2}}{2\varepsilon_{11}-1}r+\frac{p*_{1}-(1-\varepsilon_{11})}{2\varepsilon_{11}-1}(1-r)$ *(S2.2)*

$\varepsilon_{11}=\frac{1+\sqrt{1-2p*_{12}}}{2}$ *(S2.3)*

Where partial derivates of *p_1_*, refer to partial derivatives of equation (*S2.2*), using equation (*S2.3*) and applying the chain rule. Additionally, note that two covariance terms and are zero (and thus not included in equation (*S2.1)*) because the individuals classified only once (*y*) are independent of those classified twice (*z*).

We can now apply the following facts about the variance and covariance of estimators from multinomial distributions:

$Var(\hat{p}*_{1})=\frac{p*_{1}(1-p*_{1})}{(1-r)n}$ (*S2.4*)

$Var(\hat{p}*_{12})=\frac{p*_{12}(1-p*_{12})}{rn}$ (*S2.5*)

$Var(\hat{p}*_{11})=\frac{p*_{11}(1-p*_{11})}{rn}$ (*S2.6*)

(*S2.7)*

Additionally, partial derivatives are found to be:

$\frac{\partial p_{1}}{\partial p*_{1}}=\frac{1-r}{2\varepsilon_{11}-1}$ (*S2.8*)

$\frac{\partial p_{1}}{\partial p*_{11}}=\frac{r}{2\varepsilon_{11}-1}$(*S.2.9*)

$\frac{\partial p_{1}}{\partial p*_{12}}=\left(\frac{-1}{2(2\varepsilon_{11}-1)^{3}}\right)\left(\begin{matrix} -2\left(p*_{11}r+p*_{1}(1-r)\right)+2r(1-\varepsilon_{11})(2\varepsilon_{11}-1)+ \\ 2r(1-\varepsilon_{11})^{2}+(1-r)(2\varepsilon_{11}-1)+2(1-r)(1-\varepsilon_{11}) \end{matrix}\right)$ (*S2.10*)

Substitution of (*S2.4*)-(*S2.10*) into (*S2.1*) and simplifying gives,

$Var(\hat{p}_{1})\approx\frac{1}{n(2\varepsilon_{11}-1)^{2}}\left(\begin{matrix} r\left(\begin{matrix} p*_{11}(1-p*_{11})+0.25p*_{12}(1-p*_{12}) \\ -p*_{1}(1-p*_{1})-p*_{11}p*_{12} \end{matrix}\right) \\ +\frac{1}{r}\left(\frac{p*_{12}(1-p*_{12})(1-2p*_{1})^{2}}{4(2\varepsilon_{11}-1)^{4}}\right)+ \\ \left(\begin{matrix} \frac{(p*_{11})(p*_{12})(1-2p*_{1})}{(2\varepsilon_{11}-1)^{2}}-\frac{p*_{12}(1-p*_{12})(1-2p*_{1})}{2(2\varepsilon_{11}-1)^{2}} \\ +p*_{1}(1-p*_{1}) \end{matrix}\right) \end{matrix}\right)$ *(S.2.11*)

Where we me make use of the following: (2*ε_11_*-1)^2^ = (1-2*p*_12_*) and 1-*p*_12_+*2*p*_11_*-2*p*_1_* = (2*ε_11_*-1)^2^.

Similarly, we can find an approximation of$Var(\hat{\varepsilon}_{11})$. By the Taylor series approximation, we know

(*S2.12*)

Thus,

$Var(\hat{\varepsilon}_{11})\approx\left[\frac{1}{2\sqrt{1-2p*_{12}}}\right]^{2}\frac{p*_{12}(1-p*_{12})}{rn}=\frac{p*_{12}(1-p*_{12})}{rn(4-8p*_{12})}$ (*S2.13*)
