# Supplementary material for: The Cost-Effectiveness of Reclassification Sampling for Prevalence Estimation
Source: PLoS One. 2012 Feb 13;7(2):e32058. doi: 10.1371/journal.pone.0032058 (PMC3278465; doi:10.1371/journal.pone.0032058)
Supplement: Text S3 — Finding the value of r on the range 0 to 1 that minimizes the variance of . (DOC) [file pone.0032058.s003.doc]

*Text S3*

In this section we demonstrate how derivatives can be used to find the value of *r* that minimizes the variance of $\hat{p}_{1}$. Equation (*S3.1*) shows an equation, written as a function of *r*, for the variance of $\hat{p}_{1}$ which substitutes equation (*7*) into equation (*6*).

$Var(\hat{p}_{1})=\left(\frac{a+c(1+r)}{B}\right)\left[rK+\frac{1}{r}L+M\right]$ (*S3.1*)

where we let

$$\begin{matrix} K=\left(\frac{p*_{11}(1-p*_{11})+0.25p*_{12}(1-p*_{12})-p*_{1}(1-p*_{1})-p*_{11}p*_{12}}{(2\varepsilon_{11}-1)^{2}}\right) \\ L=\left(\frac{p*_{12}(1-p*_{12})(1-2p*_{1})^{2}}{4(2\varepsilon_{11}-1)^{6}}\right) \\ M=\frac{1}{(2\varepsilon_{11}-1)^{2}}\left(\begin{matrix} \frac{(p*_{11})(p*_{12})(1-2p*_{1})}{(2\varepsilon_{11}-1)^{2}}-\frac{p*_{12}(1-p*_{12})(1-2p*_{1})}{2(2\varepsilon_{11}-1)^{2}} \\ +p*_{1}(1-p*_{1}) \end{matrix}\right) \end{matrix}$$

Rearranging and factoring (*S3.1*) gives (*S3.2*)

$BVar(\hat{p}_{1})=r^{2}\left(cK\right)+r\left(c\left(M+K\right)+Ka\right) +\left(c\left(M+L\right)+Ma\right)+\frac{L\left(a+c\right)}{r}$ (*S3.2*)

We can find the value of *r* that minimizes the variance by taking derivatives (equation *S3.3*) and using the cubic formula to find roots in the range of *r* from 0 to 1. The optimal value of *r* will either occur at one of these roots, at *r*=1, or at the smallest practical value of *r*. We note that since *(S3.3)* does not depend on *B* (and, hence, not on *n*) sample size does not impact the optimal value of *r*.

(*S3.3*)
