# Supplementary material for: The Cost-Effectiveness of Reclassification Sampling for Prevalence Estimation
Source: PLoS One. 2012 Feb 13;7(2):e32058. doi: 10.1371/journal.pone.0032058 (PMC3278465; doi:10.1371/journal.pone.0032058)
Supplement: Text S4 — Variance for two-phase and one-phase sampling studies. (DOC) [file pone.0032058.s004.doc]

*Text S4*

McNamee (2003) gives the variance of the prevalence estimate for an optimally designed two-phase study. Translating to our notation gives (*S4.1*).

$Var(\hat{p}_{1})=$ $\frac{1}{B}\left(\sqrt{c_{g}}\left(\sum_{i=1}^{2} \left(p*_{i}\sqrt{\frac{p_{1}\varepsilon_{1i}}{p*_{i}}\left(1-\frac{p_{1}\varepsilon_{1i}}{p*_{i}}\right)}\right)+\sqrt{c+a}\left(\sqrt{\sum_{i=1}^{2} \left(\frac{(p_{1}\varepsilon_{1i})^{2}}{p*_{i}}\right)-p_{1}^{2}}\right)\right)\right)^{2}$ (*S4.1*)

We note that McNamee (2003) considers an optimally designed two-phase study to use the gold standard classification strategy at *different rates* depending on classification at the first stage. For example, 50% of people classified as diseased using the cheaper classifier might get the gold standard compared to only 10% of people classified to not have the disease. Equations to determine what percent of individuals should be given the gold standard in each group can be found in McNamee. We note that while, as described in McNamee in many cases, the equation for variance in (*S4.1*) is correct, for some situations this equation needs to be modified (see McNamee for details). We follow McNamee’s guidance to optimally design the two-phase sampling design using either the variance equation in (*S4.1*) or an alternative as provided by McNamee.

The variance of the prevalence estimate for the one-phase design is simply

$Var(\hat{p}_{1})=\frac{c_{g}p_{1}(1-p_{1})}{B}$ (*S4.2*)

since *n=B/c_g_* and this is merely a Simple Random Sample (SRS) using the gold standard for all classifications.
